# Burns From Hot Wheat Bags: A Public Safety Issue

**Published:** 2011-08-19

**Authors:** Anna Collins, Mathew Amprayil, Nicholas S. Solanki, John Edward Greenwood

**Affiliations:** ^a^School of Medicine, University of Adelaide, Adelaide, South Australia 5000, Australia; ^b^Adult Burns Unit, Royal Adelaide Hospital, North Terrace, Adelaide, South Australia 5000, Australia

## Abstract

**Introduction:** Wheat bags are therapeutic devices that are heated in microwaves and commonly used to provide relief from muscle and joint pain. The Royal Adelaide Hospital Burns Unit has observed a number of patients with significant burn injuries resulting from their use. Despite their dangers, the products come with limited safety information. **Methods:** Data were collected from the Burns Unit database for all patients admitted with burns due to hot wheat bags from 2004 to 2009. This was analyzed to determine the severity of the burn injury and identify any predisposing factors. An experimental study was performed to measure the temperature of wheat bags when heated to determine their potential for causing thermal injury. **Results:** 11 patients were admitted with burns due to hot wheat bags. The median age was 52 years and the mean total body surface area was 1.1%. All burns were either deep dermal (45.5%) or full thickness (54.5%). Ten patients required operative management. Predisposing factors (eg, neuropathy) to thermal injury were identified in 7 patients. The experimental study showed that hot wheat bags reached temperatures of 57.3°C (135.1°F) when heated according to instructions, 63.3°C (145.9°F) in a 1000 W microwave and 69.6°C (157.3°F) on reheating. **Conclusions:** Hot wheat bags cause serious burn injury. When heated improperly, they can reach temperatures high enough to cause epidermal necrosis in a short period of time. Patients with impaired temperature sensation are particularly at risk. There should be greater public awareness of the dangers of wheat bag use and more specific safety warnings on the products.

Wheat bags are therapeutic devices consisting of a fabric casing filled with wheat and fragrant oils. They are readily available in Australia, New Zealand, and the United Kingdom. They are often sold over the Internet with manufacturers and distributors established in the United States and Canada but are also commonly stocked in many pharmacies and department stores. After heating in a microwave, they are commonly used to provide relief from abdominal, joint, and muscle pain and provide warmth during winter.^1^ Used inappropriately, they have the potential to cause burn injury. This has been observed at the Royal Adelaide Hospital (RAH) Burns Unit, where a small number of significant injuries have been seen resulting from their use. This prompted a media campaign in August 2009 in an attempt to increase awareness of the dangers of overheating wheat bags. Despite having a warning label on the product, the dangers of overheating are not well known. The Australian Competition and Consumer Commission provide a safety brochure on the risks of hot water bottles but do not provide any similar information on hot wheat bags.^2^^,^^3^ A literature search revealed no articles on the dangers of wheat bags and their potential to cause serious burn injury. This was investigated by a retrospective analysis of all patients admitted to the RAH Burns Unit with burns from hot wheat bags and an experimental study to determine the temperature of various wheat bags after being heated and reheated in microwaves of differing power. This will form the basis of a public awareness campaign on the dangers of hot wheat bags.

## METHODS

### Retrospective analysis

The RAH Burns Unit database was searched for all patients admitted with burns due to “wheat bag” or “heat pack” within a 5-year period from August 2004 to August 2009. Data recorded included patient age, gender, site of burn, depth of burn, total body surface area burned, surgery required and length of hospital stay. Any predisposing factors for burn injury, such as neuropathy, were identified from the case notes.

### Experimental model

An experimental study was performed to determine the mechanisms leading to burn injuries from wheat bags given the fact that they come with safety instructions. Two causes were hypothesized that may result in the bags reaching hazardous temperatures; the wheat bags being heated in microwaves with higher power outputs than those specified in the safety instructions and the bags being reheated before they have completely cooled.

The experiment was performed in 3 parts using 3 wheat bags of different size and brand. The temperature of each bag was measured 3 times and averaged before heating to establish a starting temperature. This was performed 3 times to establish a mean overall starting temperature.

First, each wheat bag was tested in accordance with the heating instructions provided, which consisted of heating the bag in a microwave of specified power for a specified length of time. Bag 3 also advised placing a small container of water in the microwave with the bag during heating. Microwaves that corresponded as closely as possible to the power output recommended on the instructions (650 W, 700 W, 850 W) were used and each bag heated for the specified time. The temperature of each bag was then measured in 3 different areas using a precalibrated Testo 830-T1 infrared thermometer (Testo AG, Lenzkirch, Germany) with a measurement range of −30°C to +400°C and a mean temperature calculated. This was repeated on 3 separate occasions, and a mean overall temperature was calculated.

Second, each wheat bag was tested in a microwave with a power output of 1000 W to determine whether this has an effect on the temperature. This power output was chosen as it was found to be the power of the most commonly sold microwave in Adelaide after surveying 4 different department stores. The heating times given on the safety instructions for each wheat bag were used, rather than attempting to adjust the time. The temperature of each bag was measured in 3 different areas and a mean temperature was calculated. This was repeated on 3 separate occasions to calculate a mean overall temperature.

Five volunteers were asked to wear a heated wheat bag and identify when they felt it was no longer hot enough to be of benefit. The temperature of the bag was then measured 3 times and averaged. The mean temperatures from the 5 volunteers were then averaged to determine the temperature at which the bags would be reheated (the reheating temperature). Each bag was heated in a 1000 W microwave according to the instructions. The bags were then allowed to cool to the reheating temperature, before being reheated according to the instructions. The temperature of each bag was then measured 3 times and a mean temperature was calculated. This was again repeated on 3 separate occasions to calculate a mean overall temperature. The data were compiled and analyzed using Microsoft Excel 2007 (Microsoft, Redmond, California).

## RESULTS

### Retrospective analysis

Over the 5-year period studied, a total of 1913 patients were admitted to the RAH Burns Unit for a range of different types of burns (Table 1). Of these, 11 patients (0.6%) had burns due to “wheat bags.” The median age of all patients was 52 years (range, 20–72) and 64% were female. The median length of stay was 7 days (range, 1–35). The mean total body surface area was 1.1% (range, 0.5–2). The burns were either full thickness (54.5%) or deep dermal (45.5%). Areas affected were predominantly the feet (45.5%) and lower limb not including the feet (36.4%), although hands, upper limb not including the hand and back were also affected. Ten patients required operative management involving a combination of debridement, split-thickness skin grafting, and direct closure. One patient required amputation of all toes on the right foot. Predisposing factors were identified in 7 patients. These included peripheral neuropathy due to diabetes mellitus, paraplegia, spina bifida, and spinal block during caesarean section. These results are listed in Table 2.

### Experimental model

All 3 wheat bags had a starting temperature between 22.0°C and 23.5°C (71.6°F-74.3°F). When heated according to safety instructions, the mean temperatures were 57.2°C (135.0°F) [range, 55.0°C-60.9°C (131.0°F-141.6°F)] for bag 1, 57.3°C (135.1°F) [range, 54.0°C-60.0°C (129.2°F-140.0°F)] for bag 2, and 46.8°C (116.2°F) [range, 45.5°C-48.0°C (113.9°F-118.4°F)] for bag 3.

On heating the wheat bags in a 1000 W microwave, the maximum temperature reached was 65.0°C (149.0°F). Mean temperatures were 61.4°C (142.5°F) [range, 57.0°C-64.0°C (134.6°F-147.2°F)] for bag 1, 63.3°C (146.0°F) [range, 62.0°C-65.0°C (143.6°F-149.0°F)] for bag 2, and 37.5°C (99.5°F) [range, 35.0°C-39.0°C (95.0°F-102.2°F)] for bag 3. The temperatures of wheat bags 1 and 2 were consistently higher than those reached in the first part of the experiment.

**Figure 1:**
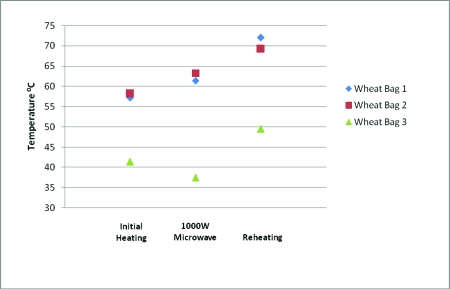
Comparison of wheat bag temperatures when heated.

When the wheat bags were reheated, the mean temperatures were 68.8°C (155.8°F) [range, 65.0°C-75.0°C (149.0°F-167.0°F)] for bag 1, 69.6°C (157.3°F) [range, 68.5°C-70.5°C (155.3°F-158.9°F)] for bag 2, and 48.5°C (119.3°F) [range, 46.5°C-50.5°C (115.7°F-122.9°F)] for bag 3. These results are illustrated in Table 3 and Figure 1.

## DISCUSSION

A skin surface temperature of 44°C (111.2°F) can cause complete epidermal necrosis after a period of 6 hours.^4^ Above this temperature, the time taken for complete epidermal necrosis to occur rapidly decreases as temperature rises. In a study by Moritz et al,^4^ the shortest time to cause complete epidermal necrosis at 49°C (120.2°F) was 9 minutes, at 55°C (131.0°F) was 30 seconds, and at 60°C (140.0°F) was 5 seconds.

This study demonstrates that wheat bags can reach hazardous temperatures. Wheat bags 1 and 2 reached higher temperatures than wheat bag 3. This is presumably due to the effect of placing a container of water in the microwave during heating as specified in the instructions. After reheating, all 3 wheat bags reached temperatures greater than 50°C (122.0°F), which can cause burns within a short period of time.

Although the wheat bags tested had heating and reheating instructions, these were only specified for certain power outputs. This is impractical as many people do not have a microwave of corresponding power and are unlikely to know how to adjust heating time accordingly. Domestic microwaves are typically 1000 W, which can heat wheat bags to hazardous temperatures between 60.0°C and 65.0°C (140.0°F–149.0°F).

Repetitive heating before the wheat bag is adequately cooled led to even greater temperatures. The maximum reheating temperature recorded was 75.0°C (167.0°F) across the 3 bags, with 2 bags reaching temperatures in excess of 68.5°C (155.3°F). Epidermal necrosis occurs in less than 2 seconds at this temperature.^4^

In the general population, hot wheat bags are unlikely to cause burn injury, as the increased temperature would alert the user to remove the bag before significant injury occurs. However, patients who have impaired temperature sensation may not detect the increased temperature and leave the wheat bag on for a prolonged period of time. This is particularly prevalent in patients with diabetes who suffer from neuropathy.

There have also been reports of wheat bags causing fires and flame injuries when overheated. This is attributed to super-heating of wheat bags in the microwave leading to a smoldering process in which temperatures may exceed 700°C (1292.0°F).^5^

Another factor contributing to wheat bag burns could be a lack of public awareness. As these are not a common cause of burns, comprising only 0.6% of the total admissions to the Burn Centre during this 5-year period, there have been comparatively few public safety initiatives. This may account for disregard for safety instructions when it comes to heating and reheating wheat bags. There is also insufficient warning on wheat bag labels and packaging regarding the risk of overheating bags and thermal injury. A media campaign was undertaken in August 2009 involving 3 radio interviews, 2 television news broadcasts, and 1 newspaper article. This may have had an impact, as since then, a reduction in the number of cases has been observed. However, this may have not reached all patient demographics and so further safety campaigns are necessary. This may involve further media broadcasts and printed safety sheets distributed to general practitioners and diabetes clinics.

## CONCLUSION

The use of hot wheat bag carries a small but significant risk of serious burn injury. In the general population, if instructions are followed correctly, the risk of burn injury is small. However, in patients with impaired sensation or if the bags are heated in higher powered microwaves or reheated, this risk is increased. A public awareness campaign in 2009 may have contributed to the reduction in burns from hot wheat bags; however, more needs to be done to prevent these injuries. It is suggested that wheat bags carry a specific warning that incorrect or excessive heating and prolonged skin contact can cause serious burns. Product labels should contain heating instructions for microwaves of different power outputs. In addition, they should be used with caution in patients with impaired sensation. There is a need for further public awareness campaigns, which could involve media pathways such as newspapers or current affairs programs. A safety sheet by the Australian Competition & Consumer Commission, similar to the safety alert for hot water bottles could be produced and distributed to places where wheat bags are sold, to health care staff and to the general public.

## Figures and Tables

**Table 1:** Etiology of burn injury of patients admitted to the Royal Adelaide Hospital between August 2004 and August 2009

Cause of Burn Injury	Number of Patients Admitted, %
Chemical	181 (9.5%)
Contact	174 (9.1%)
Electrical	53 (2.8%)
Explosion	144 (7.5%)
Flame	549 (28.7%)
Friction	12 (0.6%)
Radiant Heat	79 (4.1%)
Scald	705 (36.9%)
Unknown	16 (0.8%)

**Table 2:** Demographic data and injury details of patients admitted with burns from hot wheat bags

Sex	Age	Predisposing Factors	Site of Burn	Depth of Burn	Total Body Surface Area, %	Treatment	Length of Stay, d
M	72	Nil identified	Foot	Deep dermal	0.5	Conservative management	6
F	49	Multiple sclerosis—spastic quadriplegia	Foot	Full thickness	1	Debridement and split-thickness skin grafting	35
M	52	Type 2 diabetes mellitus	Lower limb not including foot	Full thickness	2	Debridement and split-thickness skin grafting	12
F	64	Type 2 diabetes mellitus, L1 paraplegia	Foot	Full thickness	2	Debridement and split-thickness skin grafting	9
M	58	Nil identified	Lower limb not including foot	Deep dermal	1	Debridement and split-thickness skin grafting	9
F	20	Nil identified	Back	Deep dermal	1	Excision and primary closure	1
F	48	Type 1 diabetes mellitus	Upper limb not including hand	Full thickness	1	Debridement and split-thickness skin grafting	4
F	52	Nil identified	Lower limb not including foot	Deep dermal	1	Debridement and split-thickness skin grafting	6
F	36	Spinal block	Foot	Full thickness	0.5	Debridement and split-thickness skin grafting	7
F	29	Spina bifida	Lower limb not including foot	Full thickness	0.5	Excision and primary closure	4
M	61	Type 2 diabetes mellitus	Foot	Deep dermal	2	Debridement without skin graft	34

**Table 3:** Results of the experimental study

Wheat Bag	Dimensions cm	Time Heated,s	Power Output on Instructions	Mean Temperature When Heated According to Instructions, °C (°F)	Mean Temperature When Heated in 1000 W Microwave, °C (°F)	Mean Temperature When Reheated, °C (°F)
1	18 × 25	90	650 W	57.2 (135.0)	61.4 (142.5)	68.8 (155.8)
2	36 × 16	120	850 W	57.3 (135.1)	63.3 (145.9)	69.6 (157.3)
3	40 × 16	120	700 W	46.8 (116.2)	37.5 (99.5)	48.5 (119.3)
